# Comparison of Short-Read Sequence Aligners Indicates Strengths and Weaknesses for Biologists to Consider

**DOI:** 10.3389/fpls.2021.657240

**Published:** 2021-04-16

**Authors:** Ryan Musich, Lance Cadle-Davidson, Michael V. Osier

**Affiliations:** ^1^Thomas H. Gosnell School of Life Sciences, Rochester Institute of Technology, Rochester, NY, United States; ^2^USDA-Agricultural Research Service, Grape Genetics Research Unit, Geneva, NY, United States

**Keywords:** short-read sequencing, alignment, comparison, accuracy, runtime

## Abstract

Aligning short-read sequences is the foundational step to most genomic and transcriptomic analyses, but not all tools perform equally, and choosing among the growing body of available tools can be daunting. Here, in order to increase awareness in the research community, we discuss the merits of common algorithms and programs in a way that should be approachable to biologists with limited experience in bioinformatics. We will only in passing consider the effects of data cleanup, a precursor analysis to most alignment tools, and no consideration will be given to downstream processing of the aligned fragments. To compare aligners [Bowtie2, Burrows Wheeler Aligner (BWA), HISAT2, MUMmer4, STAR, and TopHat2], an RNA-seq dataset was used containing data from 48 geographically distinct samples of the grapevine powdery mildew fungus *Erysiphe necator*. Based on alignment rate and gene coverage, all aligners performed well with the exception of TopHat2, which HISAT2 superseded. BWA perhaps had the best performance in these metrics, except for longer transcripts (>500 bp) for which HISAT2 and STAR performed well. HISAT2 was ~3-fold faster than the next fastest aligner in runtime, which we consider a secondary factor in most alignments. At the end, this direct comparison of commonly used aligners illustrates key considerations when choosing which tool to use for the specific sequencing data and objectives. No single tool meets all needs for every user, and there are many quality aligners available.

## Introduction

Sequence aligning tools, which determine where small sequence fragments align to a larger, “reference” genome or transcriptome sequences are an essential part of any toolkit for modern whole genome and transcriptome analyses. However, the plethora of available tools (Dobin et al., 2013; Li, 2013; Kim et al., 2015) and regular addition of new tools make it difficult to decide which tool is best for the specific data set being analyzed. Even older tools can perform admirably, if not optimally, negating the mindset that “newer is better.” Therefore, being able to parse out the merits of existing, and future, tools is of great benefit to the wet lab biologist. Determining a fragment’s location in the reference allows for diverse applications, ranging from agricultural benefits like identifying how abiotic stresses can protect a crop from a fungus (Weldon et al., 2019) to discovering vulnerabilities and susceptibilities in a novel human virus such as COVID-19 (Kim et al., 2020).

To align sequenced reads, an aligner must first fragment the reference genome into smaller components. Each fragment’s location and sequence content are then stored in a data structure. The type of data structure used highly impacts an aligner’s overall runtime and memory usage. Indexing the reference genome allows for an aligner to work more efficiently by finding all exact matches to a sequenced read using a single lookup in the given data structure and not scanning the entire reference genome from start to finish for each read. As aligners have evolved over the years, the major algorithmic change that has evolved with them has been the refinement of the data structure used for indexing the reference sequence. When aligners were initially available for public use in the late 1990’s, the data structure being used to index the reference sequence was that of a suffix tree (Delcher et al., 1999). Although effective for indexing, suffix trees are known in the computing world to require a large amount of memory for their creation, with the human genome needing roughly 45 GB of space in suffix tree form (Kurtz et al., 2004). This large memory usage was a major drawback for early aligners as these tools would struggle to run on even today’s computers and would be reserved for use on research servers only. Reducing memory usage was the major goal of future tools and resulted in the use of the FM-Index as the major data structure being used by nearly all of today’s aligners.

**Figure 1:**
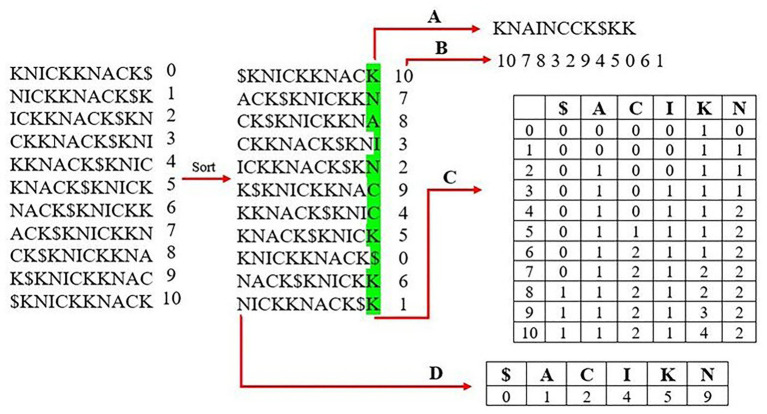
Creating an FM-index of the word “knickknack.” The first step is to generate all rotations of the reference sequence and sort them lexicographically. The last column is stored as the Burrows-Wheeler transform (BWT) in **(A)** and the corresponding suffix array in **(B)**. A rank table is created from the BWT, which lists the occurrence and order of each unique character shown in **(C)**. **(D)** shows the lookup table, which lists the index of the first occurrence of each character from the first column of the sorted matrix.

Most modern aligners use a Full-text index in Minute space, or FM-Index (Figure 1), as the genome index structure because it performs well in both overall runtime and memory usage (Ferragina and Manzini, 2000). The most important component of the FM-Index is its use of the Burrows-Wheeler transform (BWT) of the reference genome. The BWT is created by first building an array of suffix rotations that appear in the genome itself starting with the full genome as the first suffix, then the next suffix removes the first letter and appends it onto the end (i.e., the suffix rotations of the word “knickknack” are “knickknack$,” “nickknack$k,” “ickknack$kn,” and so on). The next step sorts the suffixes lexicographically, and the BWT is represented by the last column of this sorted suffix array. The BWT often contains runs in which a single character appears many times in a row due to the array having been sorted lexicographically. These runs of characters allow for compression of the BWT to further reduce the size of the resulting genome index.

**Figure 2:**
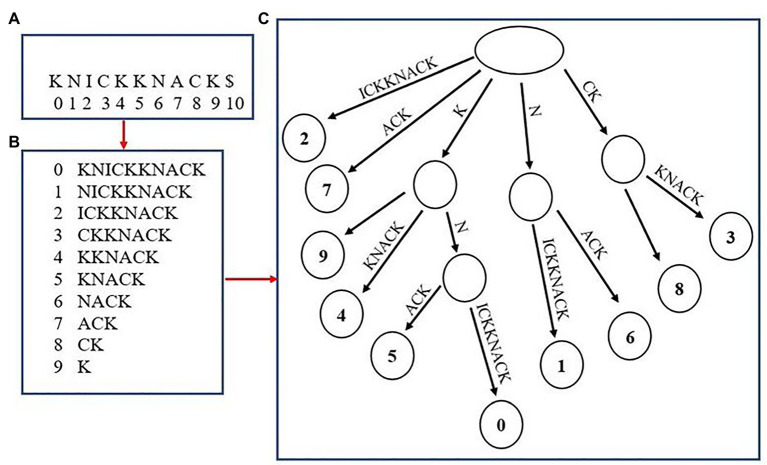
Creating a suffix array and tree from the word “knickknack.” The overall word with its associated indexes is shown in **(A)**. **(B)** shows the unsorted suffix array with their associated indexes. **(C)** shows the completed suffix tree.

Although not as common, multiple high-quality aligners use an uncompressed “suffix array” as a means to index fragments (Dobin et al., 2013; Marçais et al., 2018). Suffix arrays have been utilized since the initial introduction of genome aligners, but were used as a stepping-stone in the process of creating the original method of genome-indexing, the suffix tree (Figure 2). Some aligners today, such as MUMmer4 and STAR, have removed the extra step of creating a suffix tree and utilized aspects of the suffix array to aid in alignment. The generation of a suffix array is very similar to how the BWT suffix rotation array is created with the only exception being that the prefixes are not appended to the end of each suffix (i.e., the suffix array for the word “knickknack” is “knickknack,” “nickknack,” “ickknack,” and so on). The positions where each suffix occurs in the overall genome are recorded as well. The final step of generating the suffix array is to sort the array alphabetically, which allows for suffixes beginning with the same string of characters to appear one after the other. This allows for a fast lookup when finding exact matches of a sequenced read. For example, when finding the exact matches for a read “kn,” the algorithm will begin by finding where in the suffix array the fragment fits alphabetically, then record that there are two suffixes that begin with “kn,” “knickknack,” and “knack,” This results in the creation of two separate alignments. A benefit of using a suffix array over an FM-Index is that there is no extra step for having to convert the BWT back into the reference genome when creating alignments, which often results in suffix arrays having a faster lookup time. However, a major disadvantage to using suffix arrays is that they can require a large amount of memory depending on the size of the genome being used and may not be ideal to run on some systems.

For the end user, there are two primary considerations when deciding which aligner to use: accuracy and run time.

Accuracy of a tool can be estimated by multiple proxies. Unfortunately, it cannot be measured directly for any given experiment due to a “chicken or the egg” class of problem. To know the true accuracy of an alignment, one would need to know where in the genome each fragment is from. However, the location of each fragment is the purpose of doing the alignment in the first place. As a result, accuracy is often indirectly estimated based upon our expectations for a good and complete alignment.

Accuracy can be partially assessed by the percent of reads aligned. Most end users would prefer to extract the maximal information possible from the sequencing data, meaning aligning as many reads as possible. For high-quality sequencing with few errors and a high degree of accuracy in alignment, most sequence fragments should be assigned to a location in the reference. A large number of unaligned fragments could indicate a low accuracy of the tool. However, this assumes that the reference sequence is relatively complete, otherwise reads may be part of previously unsequenced locations in the genome or transcriptome. Unaligned reads can also result from biological complications outside of the control of the aligner. For example, “multireads” are sequences that align to multiple locations in the reference due to repetitive sequences, paralogs, or other sequence duplications. For these, the aligner is not able to determine which of the regions is the correct match, so it typically leaves the fragment out. Some aligners, such as Burrows Wheeler aligner (BWA) and STAR, will report a quantitative measure (percent and/or counts) of fragments that are multireads. Another biological cause of reduced alignment occurs when RNA transcript sequences are aligned to a reference genome, there could be poor alignment due to the inability to span splice junctions. After considering the technical and biological sources of poor alignment, a high rate of unaligned reads should give a user pause, particularly when comparing among samples of a similar type aligned to the same reference.

Accuracy is also commonly assessed by the estimated gene coverage. For an alignment of shotgun genome sequence, a low coverage of the genes despite a sufficiently large number of reads could indicate a potential systematic error in how reads are aligned. An example of such an “error” would be multireads, as discussed above. There could also be sufficient differences between the fragment and the reference, such as due to polymorphism, that a matching location is not identified. Mismatch tolerance is described in more detail below. If a transcriptome is already available for the species in question, and the aligner supports the import of gene location information for annotation of alignment results, such as with a General Feature Format (GFF) or Gene Transfer Format or General Transfer Format (GTF) file, determining gene coverage is relatively facile. If gene locations for the species are not known, and a closely related species is available, it is also possible to use tools like Exonerate (Slater and Birney, 2005) and BLAST+ (Camacho et al., 2009) to perform pairwise alignments between the known genes and the aligned contigs. If the species being examined is well-studied, a tool like Benchmarking Universal Single-Copy Orthologs (BUSCO; Simão et al., 2015) can be used to count the number of core metabolic genes that exist in the resulting alignments.

Total runtime, the wall clock time a program takes to complete, is of secondary consideration for two reasons. First, in an ideal world, accuracy should be the primary consideration. Second, as seen in the section on Multithreading below, total runtime often is highly dependent on the hardware, the analysis is run on. However, with the growth in sequencing data sets over time, runtime is a non-trivial consideration that can create a research bottleneck. Total runtime is an interaction between program efficiency and the hardware upon which it is run. Since the hardware is often limited by budget constraints or institutional resources and is therefore effectively a constant for any given research group, it would be useful to know the relative efficiencies of alignment tools.

Modern software tools usually have the ability to run multiple analyses at once, or multithread. This can work similarly to how having multiple cooks in a kitchen can increase throughput up to a point. There are diminishing returns, and eventually there can be “too many cooks in the kitchen.” For a well-written program that multithreads, if there are a large number of processor cores (“cooks”) available, the “speedup” of the program should be linear with respect to the number of cores used (e.g., five cores causes the program to run five times as fast). Measuring the speedup this way (time on a certain number of cores divided by time on one core) can indicate how much faster a program could theoretically run given better hardware, accounting for the diminishing returns. In rare cases, programs can achieve “super-linear speedup,” in which the observed speedup is greater than the number of cores used. Typically, this is caused by good use of shared temporary memory or other resources. This would be similar to one cook retrieving a single container and adjacent cooks sharing it without having to spend time retrieving it themselves. As we will see later in a comparison of tools, super-linear speedup is observed in some sequence aligners.

Although the method used for indexing the reference genome impacts an aligner’s performance, so to do the parameters chosen for an individual run of the tool. Aligners typically have differences in how tolerant they are of mismatches between the fragment and the reference sequence. Some tools allow for user specification of the degree of tolerance. Typically, analyses allow one or two nucleotide differences to account for polymorphism or minor and rare sequencing errors. Even within these tolerances, some fragments will deviate too far from the reference sequence to be aligned. Note that mismatch tolerance can affect both percent alignment and gene coverage. High stringency (low tolerance) can result in a low percent alignment with too much data discarded, and a high tolerance can result in inaccuracy. Another parameter impacting downstream results is the alignment mode. Most aligners have a pre-defined mode, which is unable to be changed, while others allow the user to choose between a local or end-to-end alignment mode. In end-to-end mode, an entire read must be aligned to the reference genome for an alignment to be returned. End-to-end alignments are considered a stricter method of alignment compared to local alignments. In local mode, bases are able to be trimmed from either the 5' or 3' end of a read for the best possible alignment to be returned. This extra trimming step often causes the runtime for local alignments to be longer than an end-to-end alignment; however, the leniency of the alignment allows for higher alignment rates. Another impactful parameter for RNA-seq aligners, such as HISAT2, STAR, and TopHat2, is the option of inputting a transcriptome alongside the reference to aid in an aligner’s ability to identify potential splice sites when aligning RNA reads. Other aligners were created to mainly handle alignment of DNA reads to a reference genome, but can map RNA reads by allowing for large gaps in the resulting alignments. In this assessment, all aligners were run without the use of a transcriptome to compare performance more equitably.

In order to increase awareness among researchers of the differences between alignment programs, and to give researchers a framework for evaluating those differences, we compare a number of commonly used alignment programs in reference to the above considerations. Knowing how to choose the best tool for a particular research problem can improve the quality of downstream analyses.

## Materials and Methods

### Sequence Generation

As a reference data set, 48 samples of geographically diverse *Erysiphe necator*, or grapevine powdery mildew, were isolated and sequenced. RNA was isolated from the fungal samples by using clear nail polish on the infected leaves to separate the fungal tissue from the leaves followed by RNA extraction (Cadle-Davidson et al., 2009). Samples were sequenced in one single-end run of an Illumina GA HiSeq with five base-pair barcodes provided for each isolate. The pooled library of reads was run through the barcode splitter of the FASTX-toolkit (v0.0.13; Hannon, 2010) to create a separate file of reads for each of the 48 isolates. Each sample was run through a quality control and cleanup pipeline consisting of FastQC v0.11.7 (Andrews, 2018) and the FASTX-Toolkit.

### Alignment of Short-Reads

For all aligners, the reference genome of the C-strain of *E. necator* (Jones et al., 2014) was indexed to reduce any effects on runtime due to differences in indexing methods. All 48 samples were then aligned to the *E. necator* reference genome using the default settings of the following tools: Bowtie2 v2.3.5.1 (Langmead and Salzberg, 2012), BWA v0.7.17-r1188 (Li, 2013), Hierarchical Indexing for Spliced Alignment of Transcripts v2.1.0 (HISAT2; Kim et al., 2015), MUMmer4 v4.0.0beta2 (Marçais et al., 2018), Spliced Transcripts Alignment to a Reference v2.5.4b (STAR; Dobin et al., 2013), and TopHat2 v2.1.1 (Kim et al., 2013).

### Alignment Metrics

Where available, internal reports of the percent of aligned reads were used. For BWA and MUMmer4, samtools v1.6 (Li et al., 2009) was used to generate a BAM file from which aligned reads could be counted. Multireads were handled using the default method for each aligner. To identify the transcriptome coverage of the alignments, the Cufflinks v2.2.1 (Trapnell et al., 2010) package was used to combine alignments across all samples into FASTA files using the cufflinks and cuffmerge tools. BLAST+ v2.5.0+ (Camacho et al., 2009) was then used to make a database of the compiled alignment files. A blastn search was performed with the reference transcriptome (Jones et al., 2014) as the query sequences. The result of each BLAST+ run was a report of the single-best alignment per *E. necator* transcript if it was found in the pre-built aligner’s database. Transcriptome coverage for a given aligner was calculated by dividing the total number of alignments found by the total number of transcripts in the transcriptome.

Sequences from the reference transcriptome that did not have BLAST alignments were collected in a FASTA file and submitted to EggNOG-mapper v2 (Huerta-Cepas et al., 2017). The proportion of missing genes, which were reported to belong to each Clusters of Orthologous Groups of proteins (COG) category assigned by EggNOG-mapper (Tatusov et al., 2000) was calculated.

### Analysis of Speed and Parallelization

Alignments were run on a dedicated dual Xeon E5-2643 (six cores and 12 threads for each processor) with 512 GB of RAM and timed as CPU time (user + system). Only one analysis was run at a time. The final runtime for each sample was normalized by the number of reads in that sample, as samples with higher read counts take longer to process than those with lower read counts. Runtime per read was averaged across all samples.

The fraction of code that can be parallelized (F_par_) was estimated from the speedup (S) of runtime from 1 processor to n processors using a rearrangement of Amdahl’s law [F_par_ = (1/S−1)/(1/n−1)]. The F_par_ values for 2, 3, and 5 processors were averaged, and the maximum theoretical speedup calculated by Amdahl’s law for infinite processors as 1/(1−F_par_).

## Results

To compare some commonly used aligners, an RNA-seq dataset of *E. necator* was used. Although aligners may show different results with another species, *E. necator* was selected here due to extensive knowledge on the species through years of research. Each sample was run through a quality control and cleanup pipeline, and then aligned to the reference genome. One of the alignment tools tested, Bowtie2, has two distinct alignment modes (End-to-End and Local), so both modes were tested to examine if one was better than the other. Additionally, TopHat2 is no longer supported and has been replaced with HISAT2 but is included for reference.

**Figure 3:**
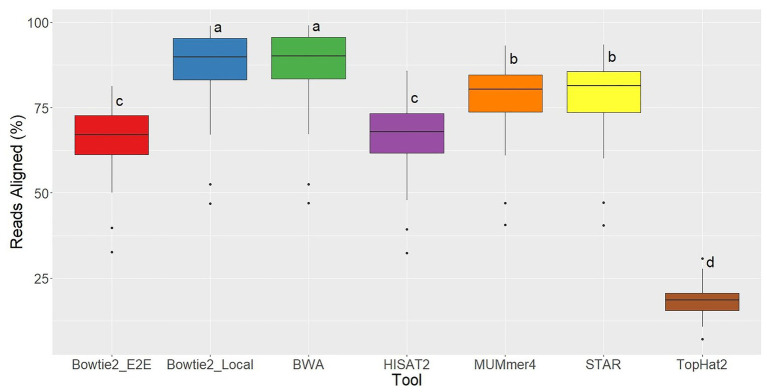
Alignment rate distributions for each of the seven aligners tested across 48 RNA-seq samples. Aligners with the same character above the boxplots were not significantly different based on Tukey’s HSD with 95% confidence level.

For each of the 48 samples, the alignment rate was tracked for all aligners used, which represents the percentage of sequenced reads that were successfully mapped to the reference genome. TopHat2 returned the lowest alignment rates by far, demonstrating the significant improvements in alignment quality in this newer generation of tools. Bowtie2 in End-to-End alignment mode and HISAT2 had similar alignment rates (averages = 66%; Figure 3). Bowtie2 run in Local alignment mode and BWA achieved the highest alignment rates (averages = 87%), and MUMmer4 and STAR were intermediate (averages = 78%; Figure 1).

**Figure 4:**
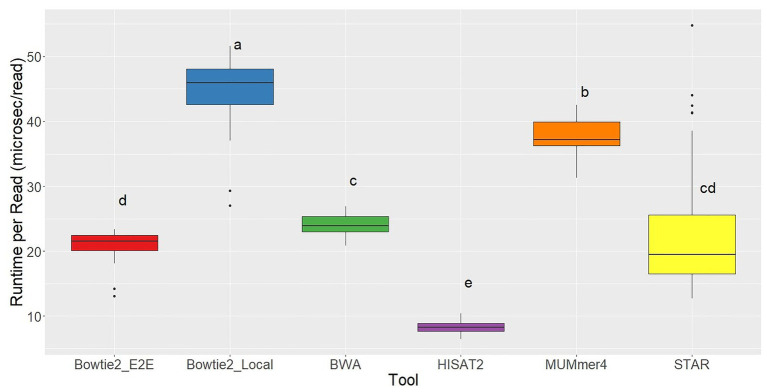
Runtime per read distributions for each of the seven aligners tested across 48 RNA-seq samples. Aligners with the same character above the boxplots were not significantly different based on Tukey’s HSD with 95% confidence level.

Running each of the aligners using five cores, the slowest tool by far was TopHat2 with an average runtime of 221.07 ms per read, 5x slower than the next slowest aligner. This again clearly demonstrates the significant improvements in alignment speed. The fastest aligner tested was HISAT2, which recorded a runtime of 8.28 ms per read on average (Figure 4).

Additionally, aligners were assessed on how well they were able to be parallelized by using multiple computational cores. We define speed-up as the time required using one core divided by the time required on X cores. Most aligners tested achieved linear speedup, such as Bowtie2 in Local mode (slope = 0.999), BWA (slope = 0.833), HISAT2 (slope = 0.876), and MUMmer4 (slope = 0.981). Bowtie2 in End-to-End mode showed slight super-linear speedup (slope = 1.059). Both STAR and TopHat2 had logarithmic speedup curves. Using Amdahl’s Law for calculations, the maximum theoretical speedup for STAR and TopHat2 were found to be 6.17 and 3.10, respectively.

**Figure 5:**
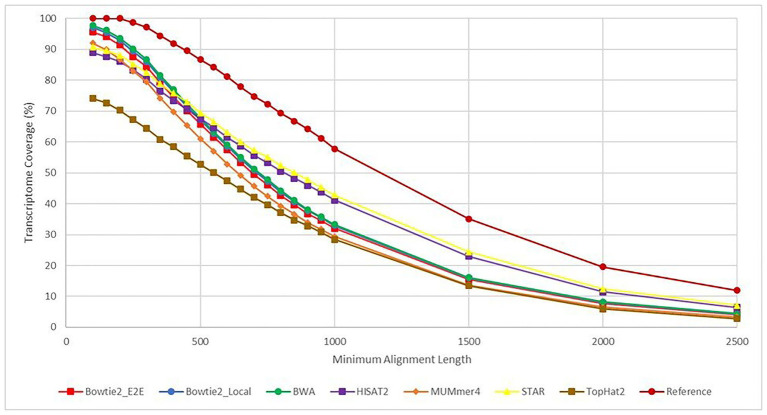
Transcriptome coverage based on a minimum alignment length cutoff. This figure shows the transcriptome coverage for each aligner calculated from the BLAST+ alignment results. The *y*-axis shows the transcriptome coverage calculated for varying alignment length cutoffs ranging from 100 to 2,500 base pairs (*x*-axis). Coverage for an aligner was calculated taking the total number of alignments returned by BLAST+ with length greater than or equal to the cutoff value and dividing by the total number of transcripts in the *Erysiphe necator* reference transcriptome. Each line is a different aligner except for the “Reference” plot, which represents the percent of transcripts from the reference transcriptome that follow the alignment length cutoffs. This line represents the theoretical maximum for each cutoff value.

The results from each aligner were then analyzed to determine how much of the overall *E. necator* transcriptome was covered based on the alignment results for all 48 samples. Because each of the samples came from a geographically distinct area, representing the genetic diversity of the species, combining the results for all 48 samples was performed with cufflinks and cuffmerge to remove the bias of any sample not expressing a particular gene. In this sense, we analyzed the maximal transcriptome coverage an aligner can achieve given 48 replicates. To further analyze whether some aligners were better at mapping longer or shorter transcripts, the transcriptome coverage was calculated at various alignment length cutoffs (Figure 5). Overall, all aligners except TopHat2achieved ~90% or greater transcriptome coverage using alignments that were at least 100 bases long. BWA had the highest recorded coverage at this cutoff at 97.8% followed closely by Bowtie2 in both Local (97.1%) and End-to-End mode (95.6%). As the alignment length cutoff value was increased, the coverage values for most aligners converged to the same values. However, both HISAT2 and STAR were able to achieve higher coverage values for alignments >1,000 bases signifying that these two tools are better at mapping larger transcripts than the other aligners tested.

Aligners were further checked using eggNOG-mapper v2 (Huerta-Cepas et al., 2017) for any bias as to the types of genes, which were unable to be mapped to the reference genome. Overall, all aligners tested had similar distributions between COG categories with categories “L” (“Replication, Recombination, and Repair”) and “S” (“Function Unknown”) being overrepresented in each (Table 1). The lowest number of unmapped genes was 106 from BWA with ~20% of these genes belonging to the “Replication, Recombination, and Repair” category and ~12% belonging to “Function Unknown.” The highest number of unmapped reads belonged to TopHat2 with a total number of 1,570 unmapped genes resulting in ~12% belonging to “Replication, Recombination, and Repair” and ~29% belonging to “Function Unknown.” Interestingly, as the number of unmapped genes increased between aligners, the percentage of genes belonging to “Replication, Recombination, and Repair” steadily decreased and the percentage of genes belonging to “Function Unknown” steadily increased.

**Table 1:** Distribution of unmapped transcripts from each aligner into EggNOG functional annotation categories.

	BWA	Bowtie E2E	STAR	TopHat2
	*n* = 106 (%)	*n* = 242 (%)	*n* = 532 (%)	*n* = 1,570 (%)
A. RNA processing and modification	1.9	4.1	2.4	3.4
B. Chromatin structure and dynamics	0	0	1.3	1.5
C. Energy production and conversion	0.9	1.2	0.6	1.0
D. Cell cycle control, cell division, chromosome partitioning	0	0.8	1.5	2.4
E. Amino acid transport and metabolism	0.9	0.4	0.6	1.9
F. Nucleotide transport and metabolism	0.9	0.4	0.6	0.8
G. Carbohydrate transport and metabolism	0	0	0.2	0.9
H. Coenzyme transport and metabolism	0	0.4	1.1	1.3
I. Lipid transport and metabolism	0.9	0.8	0.8	1.0
J. Translation, ribosomal structure, and biogenesis	1.9	1.6	1.7	2.4
K. Transcription	0	1.2	2.4	2.0
L. Replication, recombination, and repair	20.8	15.3	15.8	12.4
M. Cell wall/membrane/envelope biogenesis	0.9	0.8	0.6	0.4
N. Cell motility	0	0	0	0.1
O. Post-translational modification, protein turnover, and chaperones	2.8	2.1	1.3	3.1
P. Inorganic ion transport and metabolism	0	0.8	0.8	1.1
Q. Secondary metabolites biosynthesis, transport, and catabolism	0.9	0.4	0.4	0.6
R. General function prediction only	0	0	0	0
S. Function unknown	12.3	17.4	26.5	29.2
T. Signal transduction mechanisms	1.9	2.5	1.7	2.0
U. Intracellular trafficking, secretion, and vesicular transport	0	1.2	2.4	2.5
V. Defense mechanisms	0	0	0.4	0.3
W. Extracellular structures	0	0	0	0
X. Mobilome: prophages, transposons	0	0	0	0
Y. Nuclear structure	0	0	0	0
Z. Cytoskeleton	1.9	1.2	0.8	1.6
Uncategorized	10.4	8.3	10.5	9.7
No results	39.6	33.1	27.8	22.2

## Discussion

Based upon transcriptome coverage and alignment lengths, most commonly used tools work well. Although TopHat2 is consistently at the bottom ranking, it was included as a control as it has been superseded by HISAT2. For its generation of aligners, TopHat2 was an excellent tool. As we have seen, newer tools have made significant improvements, and nearly all have a high degree of completeness over long lengths.

An additional concern is the spanning of splice junctions when one data source (reference or aligned sequences) is genomic and the other transcriptomic. In experimentation such as RNASeq (Wang et al., 2009; Hwang et al., 2018; Stark et al., 2019), transcriptomic sequence is aligned to a genomic reference. As fragmentation previous to sequencing is usually random in location, many of the transcript sequence fragments will span a splice site. Unrecognized, this effectively creates a large gap inside the fragment with respect to the reference, which will cause the fragment to fail to align. Some aligners are designed to recognize splice junctions effectively, such as HISAT2, STAR, and TopHat2. Quantifying and comparing the capability of these tools to identify splice sites was beyond the scope of this analysis because the measures of accuracy will vary by experimental application.

Another concept to keep in mind when choosing an aligner is the type of downstream analysis required for the given research project. Nearly all aligners have the ability to output alignments in commonly used file formats, such as SAM/BAM files. In general, this means that most downstream analysis tools are compatible with the aligner’s output. However, some aligners, such as MUMmer4 in our case, output the data in an incorrect format. In our findings, each of the MUMmer4 alignment files were missing the required “@SQ” header lines (one for each contig in the reference genome), which meant that the outputted files were not able to be used for downstream analysis without some manipulation. To avoid problems like this, it is important to do the necessary research beforehand to ensure that the chosen aligner is compatible with the downstream analysis tool of choice.

It should also be noted that aligner performance can vary significantly depending on the composition of the organism’s genome or transcriptome. Larger genomes and data sets obviously will require longer runtimes, as the search space has increased. Depending on the aligner, runtimes may not increase in a linear manner, either. The degree of duplication in the genome can also complicate analysis for aligners, impacting both speed and accuracy. For example, in a genome with evolutionarily recent duplication, a given fragment may align to multiple loci. As a result, aligners may locate the fragment to multiple locations (declaring it a multiread), choose one location over others, and throw out the read as unable to be mapped, or take some other action. Similar outcomes could happen with a fragment that spans a common repetitive element. We have not examined the effects of genome complexity, including differences in the handling of multireads, on aligner performance. This would make an excellent area for future research and trials.

**Figure 6:**
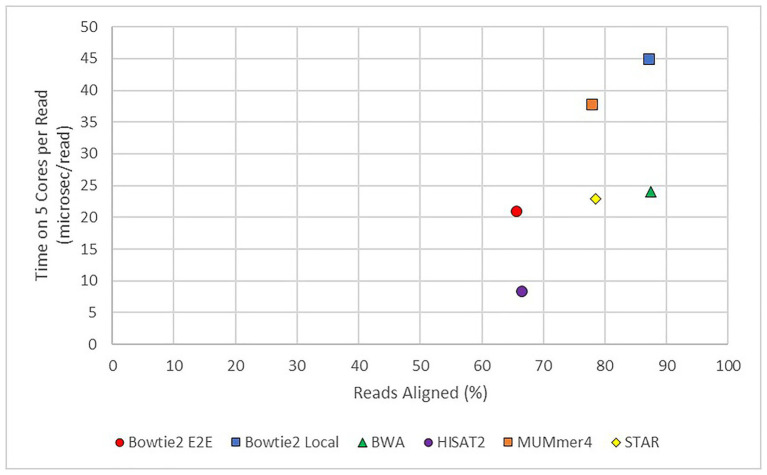
Average runtime per read vs. average alignment rate for modern aligners. TopHat2 was also used but the results were excluded from this figure as it was found to be an outlier. The *x*-axis shows the average percentage of reads successfully mapped to the *E. necator* reference genome from all 48 samples. The *y*-axis shows the average runtime per read from all 48 samples.

In practice, the choice of aligner often comes down to a trade-off between speed and completeness. As shown in Figure 6, there are significant differences in this arena. Aligners to the lower right of the graph would have the optimal mix of high speed (short runtime) and high completeness. Unfortunately, such an aligner does not exist to-date. If completeness is primary and time is not of a concern, such as a facility with excellent computational resources, an aligner such as BWA would work well. In fact, BWA still has a runtime per read in the “middle of the pack.”

If speed is the primary concern, however, a tool such as HISAT2 still has a good degree of completeness. An example of this case would be a rapid diagnostic test, such as pulsed sequence analysis (Stranneheim et al., 2014), in which rapid alignments are completed to identify possible causes of an Inborn Error of Metabolism in a critical care patient. In these experiments, fragments are aligned in periods (pulses) as they are generated by the sequencer for a first pass identification of possible nonsynonymous mutations. Other concerns are also important in this specific example, such as ability to correctly identify genetic variation from the alignment. This measurement of accuracy is outside the scope of this paper, but is of importance in a number of experimental conditions.

Although a large number of sequence aligning tools exist, most modern tools have similar key metrics and perform well for a wide variety of purposes. When resources are limited or specific features are required, the choices are often themselves more limited, and one must be more careful. As small fragment sequencing rates increase, and more fragments are generated by sequencing machines, time may become the more critical concern, too. As with the underlying sequencing technologies, alignment tool choice is a moving target. Perhaps in the future, databases and summaries of performance against gold standard data sets may be developed for specific applications. Until then, users have many fine and well established tools, and few bad choices. We hope that the above analysis and discussion helps the research community to systematically identify the alignment program that best meets their needs.

## Data Availability Statement

The names of the repository/repositories and accession number(s) can be found at: NCBI (accession: PRJNA281110, BioSamples: SAMN17885748-SAMN17885795).

## Author Contributions

All authors contributed to the research and writing of this manuscript.

### Conflict of Interest

The authors declare that the research was conducted in the absence of any commercial or financial relationships that could be construed as a potential conflict of interest.
